# Structural basis for drug-induced allosteric changes to human β-cardiac myosin motor activity

**DOI:** 10.1038/ncomms8974

**Published:** 2015-08-06

**Authors:** Donald A. Winkelmann, Eva Forgacs, Matthew T. Miller, Ann M. Stock

**Affiliations:** 1 Department of Pathology and Laboratory Medicine, Robert Wood Johnson Medical School, Rutgers University, Piscataway, New Jersey 08854, USA; 2 Department of Physiological Sciences, Eastern Virginia Medical School, Norfolk, Virginia 23507, USA; 3 Center for Advanced Biotechnology and Medicine, Robert Wood Johnson Medical School, Rutgers University, Piscataway, New Jersey 08854, USA; 4 Department of Biochemistry and Molecular Biology, Robert Wood Johnson Medical School, Rutgers University, Piscataway, New Jersey 08854, USA

## Abstract

Omecamtiv Mecarbil (OM) is a small molecule allosteric effector of cardiac myosin that is in clinical trials for treatment of systolic heart failure. A detailed kinetic analysis of cardiac myosin has shown that the drug accelerates phosphate release by shifting the equilibrium of the hydrolysis step towards products, leading to a faster transition from weak to strong actin-bound states. The structure of the human β-cardiac motor domain (cMD) with OM bound reveals a single OM-binding site nestled in a narrow cleft separating two domains of the human cMD where it interacts with the key residues that couple lever arm movement to the nucleotide state. In addition, OM induces allosteric changes in three strands of the β-sheet that provides the communication link between the actin-binding interface and the nucleotide pocket. The OM-binding interactions and allosteric changes form the structural basis for the kinetic and mechanical tuning of cardiac myosin.

## Abstract (web summary)

Omecamtiv Mecarbil (OM) is a small molecule allosteric effector of cardiac myosin in clinical trials for treatment of systolic heart failure. Here the authors determine the crystal structure of an OM-bound human β-cardiac myosin motor domain to provide molecular level insight into the mechanism of drug action.

## Graphical abstract

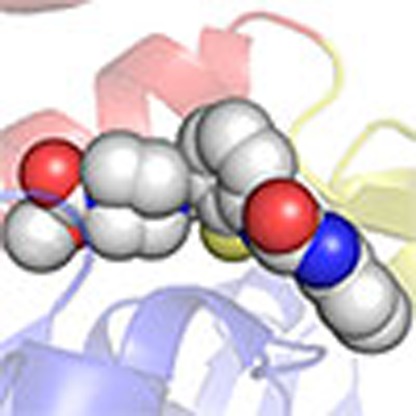

Heart failure is a common human disease with a significant lifetime risk that increases with age^1^. In its most common manifestation, heart failure is marked by a decrease in cardiac contractility culminating in systolic heart failure. Recently, a novel approach to the treatment of systolic heart failure has been developed on the basis of pharmacologic agents called ‘cardiac myosin activators' that bind directly to myosin and target the kinetic mechanism driving contraction^2^. Directly targeting the contractile mechanism of cardiac myosin could theoretically improve heart performance without altering intracellular cAMP or calcium transients. A large, high-throughput, drug screen of a biochemically reconstituted contractile system identified a series of potential cardiac myosin activators^3^. Omecamtiv Mecarbil (OM) is one such small molecule effector of cardiac myosin that is in clinical trials for treatment of systolic heart failure. Initial characterization of OM with bovine cardiac myosin shows that it binds directly to the myosin catalytic domain and operates by an allosteric mechanism to increase the transition rate of weak to strong bound actin states and enhance force generation^4^. In animal models, OM increases cardiac muscle function by extending the duration of systolic ejection without altering the heart rate^4^,^5^.

We have elucidated the precise action of OM on steady state and transient kinetics of the actomyosin mechanism and motor activity of porcine ventricular heavy meromyosin (HMM)^6^. We find that OM shifts the equilibrium of the hydrolysis step of the myosin ATPase towards products, and this accelerates the flux through the product dissociation steps of the actomyosin mechanical cycle without altering the ADP dissociation rate, the key step that regulates the maximum shortening velocity^7^. The subtle changes in the kinetic mechanism lead to an increase in the transition rate of weak to strong bound actin states, resulting in increased number of force-producing crossbridges and, paradoxically, a dramatic reduction in the unloaded shortening velocity measured *in vitro*^6^,^8^. These fascinating kinetic results led to an intense effort to determine the drug-binding site and define the structural interactions within the human β-cardiac myosin motor domain that alter the motor activity.

Purification of human cardiac myosin was not an option for accomplishing this objective because of limited availability and poor stability of clinical specimens. The alternative, expression and purification of recombinant cardiac muscle myosin, had been technically unfeasible until we showed that the principal obstacle for the expression of vertebrate striated muscle myosin was that motor domain folding follows a regulated pathway that is unique to striated muscle cells^9^,^10^,^11^,^12^. To demonstrate this principle, we designed a striated muscle myosin motor domain (MD)::green fluorescent protein (GFP) chimeric protein and expressed it in muscle cells using adenovirus-mediated expression vectors^9^. This unique single-chain motor design is functionally active, fluorescent and ideally suited for structural studies.

Here we present the structure of the human β-cardiac motor domain (cMD) fused to GFP determined without drug (Apo structure) and with OM bound (OM+ structure). OM binds in a narrow cleft that separates the N-terminal 25-K domain from the lower portion of the 50-K domain of the cMD. The drug interacts with the key residues involved in coupling structural elements that are linked to the rotation of the lever arm into the pre-power stroke conformation. Strong binding to actin triggers the reversal of the rotation, leading to the power stroke and force production. Surprisingly, OM binding induces an allosteric change in the conformation of three strands of a β-sheet that are crucial to communication between the nucleotide pocket and actin-binding interface^13^. Together, these interactions form the basis of the kinetic and mechanical effects of the drug. This structural analysis characterizes a highly significant binding cleft for drugs designed to modulate myosin motor activity.

## Results

### Human β-cardiac myosin motor domain design

The human β-cardiac myosin motor domain was expressed as a single-chain fusion protein with GFP. The protein design was based on a single-chain embryonic skeletal muscle MD::GFP chimera that was used to analyse motor domain folding^9^,^10^. The lever arm α-helix projecting from the myosin MD is truncated within the IQ motif that forms the essential light-chain-binding site and joined to a short amino terminal α-helix of GFP. This corresponds to residues 1–787 of the human β-cardiac myosin fused to residues 5–238 of *Aequorea victoria* GFP. The chimeric gene was cloned into a replication-deficient adenovirus expression vehicle, and high-titre adenovirus stocks were used to infect post-mitotic C2C12 myocytes. The brightly fluorescent cardiac MD::GFP protein was harvested from the myotubes and purified for crystallization. Nucleotide-free crystals of the β-cardiac MD::GFP were grown in hanging drops without OM (Apo structure) and with 125 μM OM (OM+ structure). SDS–polyacrylamide gel electrophoresis (SDS–PAGE) and tandem mass spectrometry (MS/MS) spectroscopy of the crystallized protein established that the full-length 117-kDa cMD::GFP chimera was crystallized (Supplementary Fig. 1).

### Structure of the Apo and OM+ cMD

**Figure 1:**
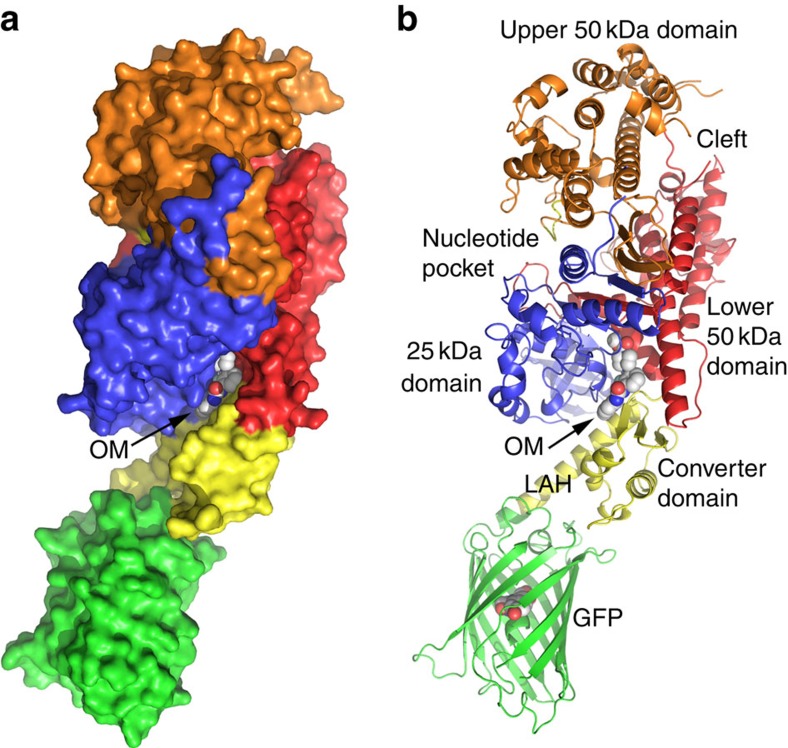
The structural organization of the human β-cardiac MD with bound OM. (**a**) Space-filling model of the A chain of the OM+ structure showing the buried OM-binding site nestled between the N-terminal 25-K domain (residues 2–204; blue) and the lower 50-K domain (residues 471–708; red). The upper 50-K domain (211–470; orange) is separated by the ‘Cleft' from the lower 50-K domain. There is no nucleotide in this structure, the cleft is ‘open' and the lever arm is extended consistent with the near-rigour conformation. The converter domain (residues 709–777) and LAH (residues 778–787) are in yellow and the GFP domain is in green. (**b**) Ribbon diagram of the same orientation to show the OM-binding site bound deeply in a narrow cleft between the 25-K and lower 50-K domains. The LAH linking the converter domain to the GFP domain is labelled. The conformation of the Apo cMD structure is very similar to the OM+ structure shown here with an r.m.s.d.=1.14 Å for all 962 Cα atoms.

The structure of the cardiac MD::GFP chimera (cMD) was determined to a resolution of 3.2 Å for the Apo and 2.25 Å for the OM+ crystals (Table 1). The drug-free Apo cMD crystallized in space group P1, and with OM bound, the protein crystallized in space group P2_1_. There are two molecules (A and B chains) in the asymmetric unit in both crystal forms, and the overall conformation of the A and B chains in each structure is very similar, independent of drug. The protein was crystallized without nucleotide and is in the extended, near-rigour conformation. The folding pattern and domain structure of the Apo and OM+ cMD structures are typical of the striated myosin II from chicken skeletal muscle root-mean-square deviation (r.m.s.d.)=0.70 Å for 558 Cα atoms), scallop striated muscle (r.m.s.d.=0.64 Å for 544 Cα atoms) and human β-cardiac myosin with AMPPNP bound (0.45 Å for 608 Cα atoms)^14^,^15^,^16^. The domains and features of the cMD structure are illustrated for the A chain of the OM+ structure in Fig. 1. The model is colour-coded to highlight the various regions of the structure associated with motor activity^15^,^17^,^18^.

The bound OM is nestled in a cleft and is just visible in the space-filling model (Fig. 1). The OM is more apparent in the ribbon diagram nestled between the 25-K domain and the lower portion of the 50-K domain. The N-terminal 25-K domain (blue) forms one face of the OM-binding cleft. About 35 Å removed from the drug cleft the 25-K domain also forms one face of the nucleotide-binding pocket. The lower 50-K domain (red) includes major elements of the actin-binding surface and forms the other face of the OM-binding cleft. The upper 50-K domain (orange) includes additional elements of the actin-binding surface that are separated by a deep cleft from the lower 50-K domain. The upper 50-K domain forms the other surface of the nucleotide pocket. The converter domain (yellow) abuts the OM-binding cleft and couples the motor domain to the lever arm helix (LAH).

In all myosin II molecules, including β-cardiac myosin, the LAH forms the light-chain-binding domain; however, in this chimeric protein the helix was truncated in the centre of the essential light-chain IQ-binding motif and fused to a short N-terminal helix of GFP (residues 791–1026; green). As a consequence, the GFP domain is positioned by design in the space normally occupied by the light chains^9^. The GFP chromophore occupies a cavity in the centre of the GFP β-barrel.

### Effect of OM on cardiac myosin motor activity

**Figure 2:**
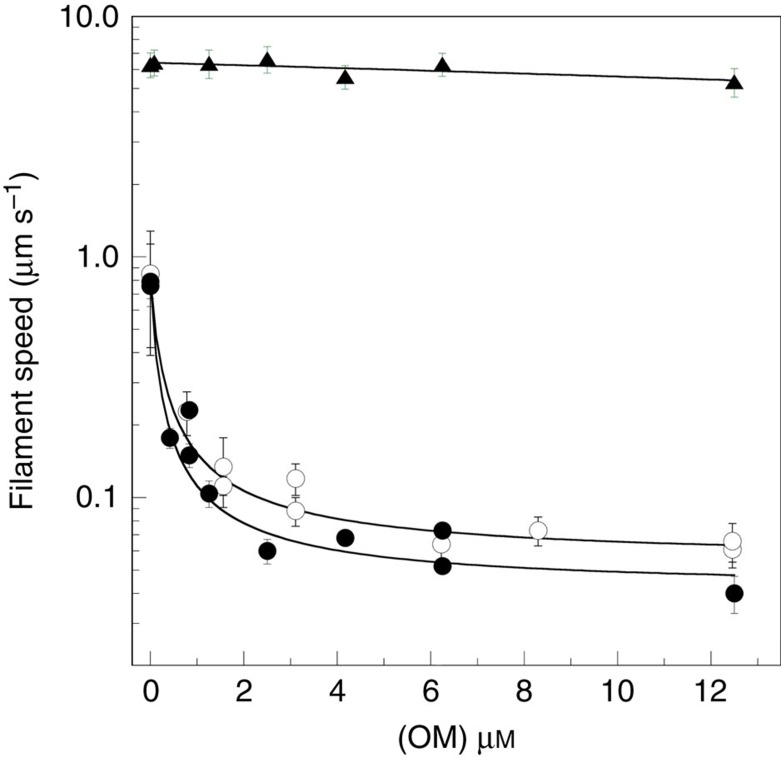
The unloaded shortening velocity of cMD is inhibited by OM. Titration of the effect of OM on the unloaded shortening velocity measured with the *in vitro* motility assay. The cMD was bound indirectly to surfaces through an anti-GFP monoclonal antibody (●) and compared with the human β-cardiac HMM (○) bound directly to the nitrocellulose surface. In the absence of the drug, both cardiac myosin fragments support actin filament velocities of 0.8 μm s^−1^. Titration with OM from 0.42 to 12.5 μM dramatically slowed the velocity of filament movement by 15–20-fold. The apparent *K*_i_ for OM binding is 100–150 nM for the human cMD and cHMM. Adult fast skeletal muscle myosin (▴) moves actin filaments at 6.3 μm s^−1^, and even the highest concentration of OM tested has little effect on filament velocity. Assays were conducted at 32 °C. The cardiac motor activity was fit to the hyperbolic inhibition curve: 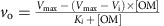; where, *v*_o_ is the observed velocity, *V*_max_ is the unloaded shortening velocity, *V*_i_ is the maximum inhibited velocity and [OM] is the concentration of drug. The mean velocity±s.d. of the velocity distribution for hundreds of filaments is plotted.

The GFP domain substitutes a rigid β-barrel for the myosin light chains and provides a convenient handle for assaying the effect of drug binding on cMD motor activity by using anti-GFP to immobilize the cMD on surfaces^9^. The unloaded shortening velocity was measured using the *in vitro* motility assay (Fig. 2). The cMD::GFP chimera supports sliding actin filament velocities of 0.8 μm s^−1^, equivalent to the human β-cardiac HMM (cHMM) and comparable to the velocity measured for porcine ventricular HMM (0.96 μm s^−1^)^6^. Addition of OM to the assay dramatically slows the filament velocity (15–20-fold) powered by both the cMD and cHMM. This is identical to what was found with porcine ventricular HMM and is explained by a drug-induced increase in crossbridges interacting with the actin filaments that impose an internal load on the sliding filament movement^6^,^8^. The *K*_i_ for OM inhibition of the sliding velocity powered by the cMD is 100 nM, indicating a high affinity for the intermediates of the mechanochemical cycle. The sliding filament velocity powered by a fast skeletal muscle myosin is much faster (6.3 μm s^−1^) than the cMD, as is expected, but is virtually unaffected by the drug, consistent with the report that OM does not bind to skeletal muscle myosin^4^.

OM modulates the steady-state ATPase of the cMD, decreasing the *V*_max_ from 5.3 to 1.9 s^−1^ but also lowering the K_ATPase_ for actin activation fourfold from 22.5 to 5.4 μM (Supplementary Fig. 2). This is consistent with the effect of OM on the porcine ventricular HMM ATPase activity. The effects of OM on the cMD motor activity and ATPase activity and the 100-nM *K*_i_ for modulating the activity corroborate the specificity of the binding site observed in the crystal structure.

### OM-binding site and the coupling region

**Figure 3:**
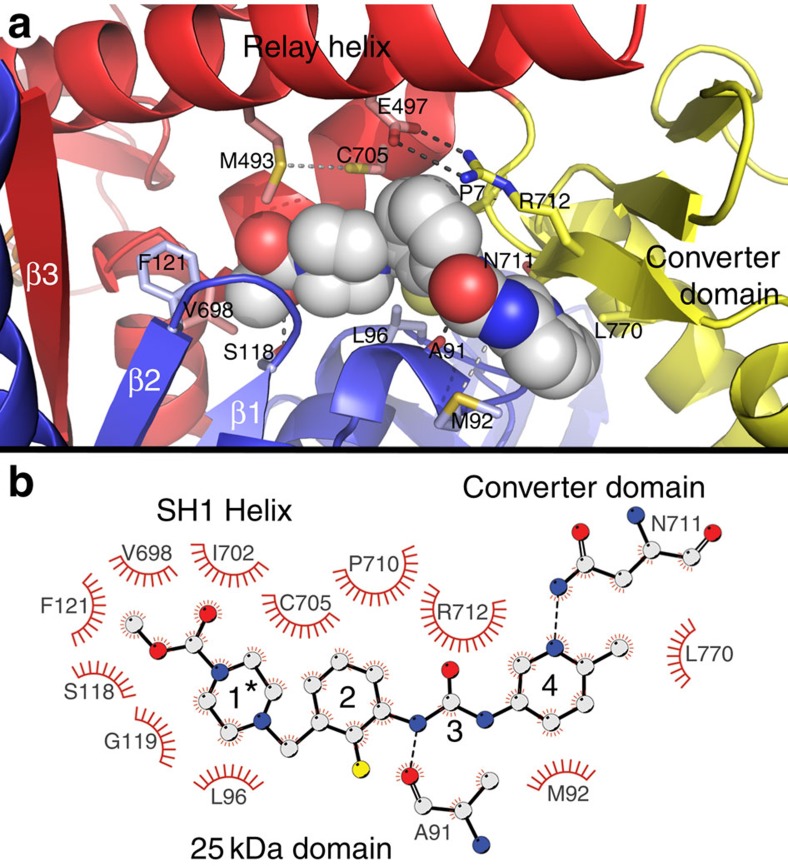
OM-binding site on the human β-cardiac MD. (**a**) A ribbon diagram of the A chain of the OM+ structure is shown with OM (space-filling model) nestled in a narrow cleft separating the 25-K domain (blue) from the lower 50-K domain (red), and interacting with residues from these domains and the converter domain (yellow). The drug is in an extended conformation with the carboxymethyl-piperazine group buried deeply in the cleft. One surface of this ring and the fluoro-benzene ring of OM make extensive packing interactions with residues along one face of the SH1 helix including C705 (SH1). The drug bridges the gap from the SH1 helix to the first strand (N711-I713) of the three-stranded β-sheet of the converter domain. In bridging the gap, OM buries a bi-dentate salt bridge, E497-R712, beneath the fluoro-benzene ring. The amido group of N711 forms a H-bond with the nitrogen of the OM methyl-pyridinyl ring. The other surface of OM wraps around a turn (A91-L96) of the 25-K domain and interacts with the first two strands (β1–β2) and connecting loop of the seven-stranded β-sheet of the cMD. The 25-K domain residues S118, A91 and M92 form likely H-bonds with OM (shown with dotted lines), and there are at least 12 packing and hydrophobic interactions as well^19^. (**b**) A protein-drug interaction diagram^64^ summarizing the most extensive interactions in the binding cleft. Hydrogen bonds involving A91 and N711 are indicated in the diagram; however, two additional H-bonds between OM and M92 and S118 are not drawn in the diagram for clarity. All of the interactions between OM and the binding cleft residues are summarized in Supplementary Table 1. *Numbers associated with the drug are added to identify the subregions of the molecule: (1) carboxymethyl-piperazine ring; (2) fluoro-benzine ring; (3) amino-carbamoyl linker; and (4) methyl-pyridinyl ring. The drug-binding interactions and the accessible and buried surface areas of the residues in the binding cleft were analysed with PISA (Protein Interfaces, Surfaces and Assemblies)^19^.

The drug, OM, is tightly nestled in a narrow cleft separating the lower 50-K domain from the N-terminal 25-K domain (Fig. 3 and Supplementary Fig. 3). OM is in an extended conformation that lies along the SH1 helix (698–707) and is adjacent to C705 (SH1) on one side and extends to and interacts with the first strand (711–713) of the three-stranded β-sheet of the converter domain. The drug packs against and wraps around two loops from the 25-K domain. OM binding involves extensive interaction with at least six residues from the 25-K domain (A91, M92, L96, S118, G119 and F121), two from the relay helix (M493 and E497), four from the SH1 helix (V698, G701, I702 and C705) and four from the β-sheets of the converter domain (P710, N711, R712 and L770; Supplementary Table 1). There are four potential hydrogen-bonding interactions between the protein and drug that involve A91, M92, S118 and N711. The N711 H-bond to the OM methyl-pyridine ring is of interest because this residue is a serine in fast skeletal muscle myosin and is one of only three sequence differences in the binding cleft that might account for the selectivity of the drug for modulating the activity of cardiac myosin but not skeletal myosin (Supplementary Table 2). The same interactions are found in the protein–drug-binding site of the A and B chains of the OM+ structure, although the conformation of the drug and binding cleft is slightly different in the two molecules. The A chain-binding site is depicted in Fig. 3.

The conformation of the main chain and the side-chain rotamers of residues lining the drug-binding cleft are essentially the same in the Apo and OM+ structures, indicating that drug binds to an existing cleft and does not induce significant local perturbation of the cMD conformation. However, analysis of the protein–drug interface indicates that OM binding results in extensive solvent shielding of residues that couple the structural elements that are central to the conformation changes of the recovery stroke: the SH1 helix, the relay helix and the converter domain. Photo-crosslinking of a benzonphenone derivative of OM had identified this cleft as the potential OM-binding site^4^. The drug-binding site is consistent with that crosslinking experiment; however, the drug is deeper in the cleft than previously imagined.

The recovery stroke involves the priming of the lever arm at the start of the mechanical cycle resulting in the rotation of the converter domain and LAH through ∼65°. The priming step is a conformational equilibrium driven by the nucleotide bound in the active site. The nucleotide state is transmitted via the relay helix to a coupling region that links the SH1 helix, relay helix and converter domain together. The packing of the drug against the SH1 helix, relay helix and converter domain shields these elements from solvent and enhances interactions among them^19^. For example, OM buries the only solvent-accessible surface of the E497-R712 salt bridge that links the relay helix to the converter domain, thereby fortifying the bond. It also shields the M493-C705 H-bond that links the relay helix to the SH1 helix (Fig. 3).

### Converter domain–LAH–GFP rotation

**Figure 4:**
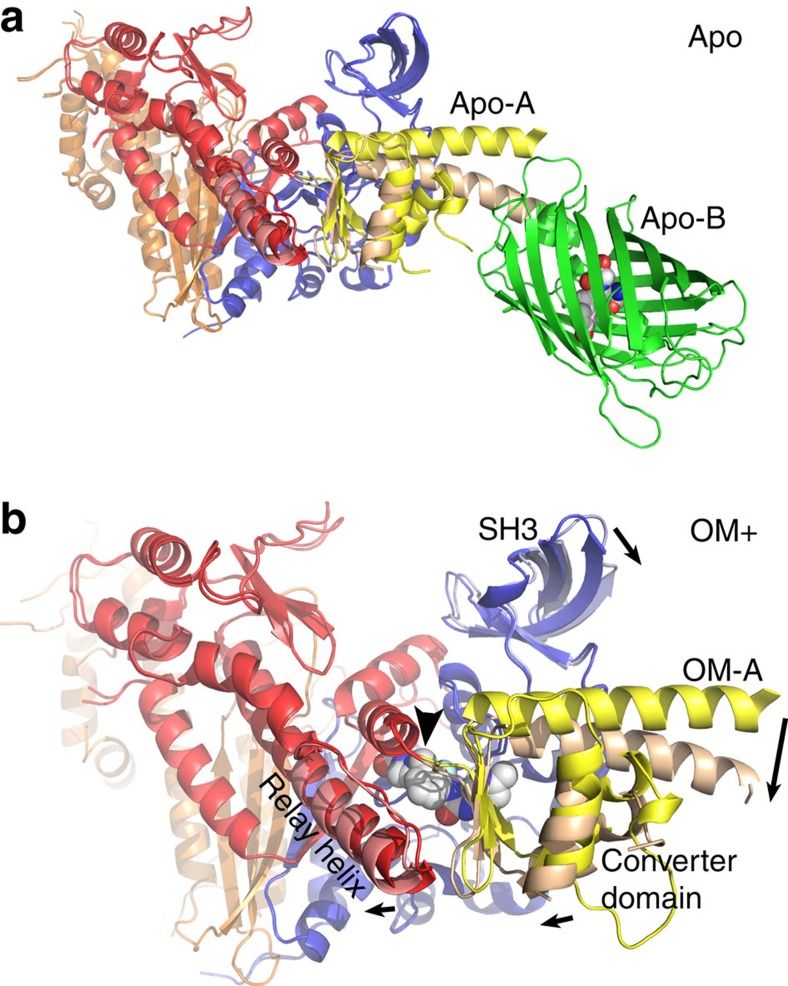
The two cardiac MD::GFP molecules in the asymmetric unit for both the Apo and the OM+ structures differ in the rotation of the LAH and GFP. In both structures, the B chain converter domains, LAH and GFP, are rotated ∼15° from the near-rigour configuration towards the pre-power stroke state. (**a**) The core MD (residues 2–708) of the Apo-A and B chains is aligned to show the rotation of the B chain converter domain and LAH (coloured tan). The A chain GFP domain is not shown for clarity. (**b**) The A and B chains of OM+ structure are similarly aligned and enlarged to show the rotation of the LAH. The A chain OM molecule is shown as a space-filling model in the centre of the image. The B chain converter domain and LAH are coloured tan. The arrows indicate the direction of rotation of the SH3-like domain, the LAH, the converter domain and the relay helix of the B chain relative to the A chain. The point of rotation in both structures is G708, immediately above OM and marked by an arrowhead in **b**. Perhaps as a consequence of the rotation, the B chain converter domains, LAH and GFP, are less well defined in both structures with higher crystallographic atomic displacement parameters.

There are two distinct molecules in the unit cell (A and B) of both the Apo and OM+ structures. In both structures the core motor domains (residues 2–708) are packed tightly together in the crystal lattice with extensive non-crystallographic symmetry between them. The conformation of the core motor domain is very similar in the two molecules of the Apo (r.m.s.d.=0.305 Å for 567 Cα atoms) and the two molecules of the OM+ (r.m.s.d.=0.311 Å for 550 Cα atoms) structures. The similarity is shared between corresponding core motor domains of the Apo and OM+ in pairwise comparison (Apo-A to OM-A r.m.s.d.=0.33 Å; Apo-B to OM-B r.m.s.d.=0.36 Å). The organization of the converter domains, the position of the GFP domains and the LAH linking the two are markedly different between the A and B chains in the asymmetric unit of both crystal structures. There is an ∼15° rotation of the converter domain–LAH–GFP domain as a unit between the two molecules in the unit cell of both crystal structures (Fig. 4). The apparent 15° rotation of the LAH from the A to the B position is in the same direction as a much larger LAH rotation during the recovery stroke (∼65°). The conformation change involves movement of the relay helix and a portion of the N-terminal 25-K region called the SH3 domain in addition to the converter domain–LAH. The hinge point for the rotation is G708 of the cMD, adjacent to the OM-binding cleft. As a consequence, the conformation of the drug-binding cleft is different between the OM+ A and B chains.

### OM moves with the rotation of the converter domain

**Figure 5:**
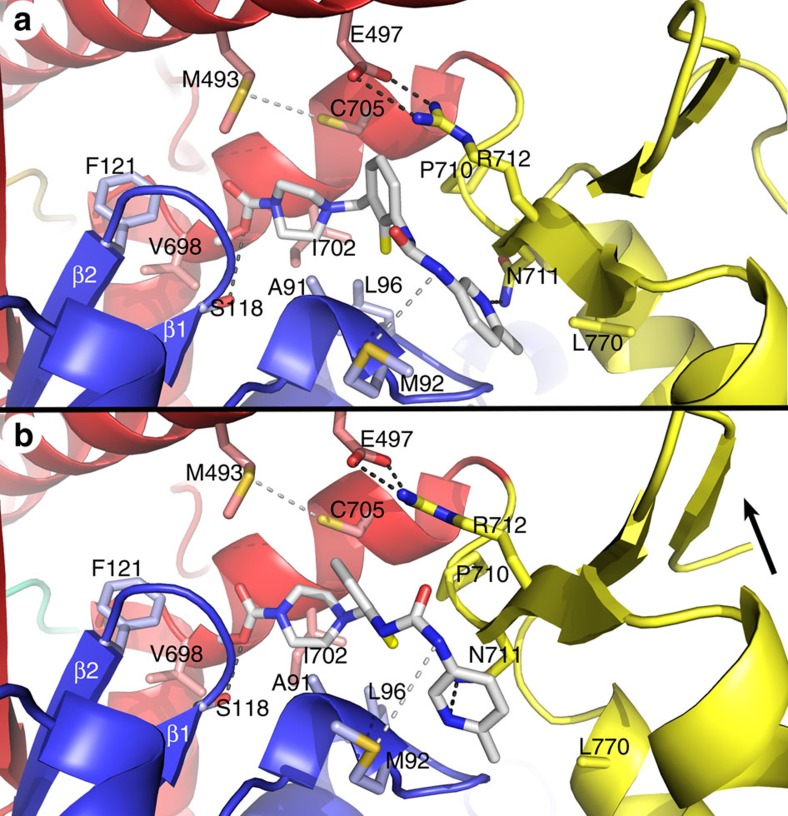
The differences in the drug-binding cleft between the two molecules in the OM+ structure. (**a**) The A chain conformation of the drug-binding site showing the interacting residues. (**b**) The B chain conformation of the drug-binding site. OM interacts with the same set of residues in both conformations of the binding cleft. The principal change between OM-A and OM-B is the rotation of the converter domain indicated by the arrow in **b**. In this view the converter domain moves up and towards the viewer, shifting the position of both N711 and R712. The fluoro-benzyl and methy-pyidinyl rings of OM rotate between OM-A and OM-B conformations as does the amino-carbamoyl group linking them. This maintains the H-bond to N711 and the shielding of the E497-R712 salt bridge. The carboxymethyl-piperazine is buried deep in the cleft and interacts with S118 and F121 of the β1 and β2 strands of the seven-stranded β-sheet.

The changes in the drug cleft as a consequence of the 15° rotation of the converter domain–LAH in the two molecules of the OM+ structure (OM-A and OM-B) are shown in Fig. 5. To accommodate changes in the position of the converter domain, there is rotation of the fluoro-benzene and methyl-pyridinyl rings and the amino-carbamoyl linker of OM. These changes mirror the shift in position of the residues interacting with the drug. In particular, the bond distance for the H-bond with N711 remains constant despite significant movement of the β-strands of the converter domain. OM follows the linkage between the SH1 helix and converter domain–LAH elements in the two molecules (OM-A and OM-B). The carboxymethyl-piperazine ring of OM is most deeply buried in the drug-binding cleft and changes very little between the two conformations of the cleft. This OM ring interacts with residues of strands β1 and β2 of the central seven-stranded β-sheet of the cMD structure. Comparison of the Apo and OM+ structures reveals subtle changes in the seven-stranded β-sheet that propagate out from the drug-binding cleft, perhaps arising from the interaction with these first two strands.

### The transducer region

Twisting and distortion of the last three strands (β5, β6 and β7) of the seven-stranded β-sheet of the myosin MD is essential for rearrangements within the nucleotide-binding pocket depending on the nucleotide state and forms the structural basis for communication between the actin-binding interface, the nucleotide pocket and the converter domain^13^,^20^. The structural elements have been called collectively ‘The Transducer' and include Loop 1 (204–210), the β-bulge (252–259) connecting strands β6 and β7 and the loop-linking helix O (HO) to the β5-strand (448–455). The β5-strand leads directly into Switch II (SW-II): the γ-phosphate-sensing loop in the nucleotide pocket. Switch II in turn leads into the relay helix, the coupling region and the drug-binding cleft.

**Figure 6:**
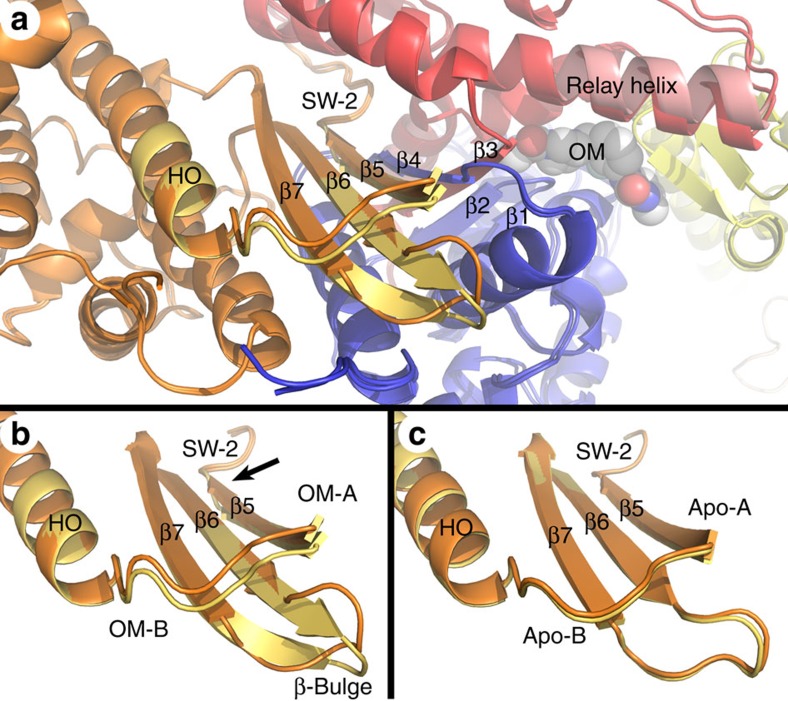
Conformational changes in the Transducer region in the OM+ structure. (**a**) The core cMD (2–708) of the OM-A and OM-B chains were aligned. Overall, the conformations of the two chains are nearly identical (r.m.s.d.=0.31 Å); however, strands β5, β6 and β7 of the β-sheet and the elements of the Transducer region (the β-bulge and HO-β5 loop) are significantly different. The OM-A chain in this region is coloured orange and the OM-B chain is yellow-orange. Displacement of the relay helix as a result of LAH rotation is also highlighted. Note that OM (space-filling model) in the binding cleft is 35 Å from the Transducer elements. (**b**) Comparison of the isolated Transducer elements of OM-A (orange) and OM-B (yellow-orange) showing an increased twist, a downward shift of the HO-β5 loop and extension of the β6 and β7 strands of the sheet into the β-bulge. The arrow highlights the changes in β5 to SW-II link. These differences are between the two molecules in the same unit cell. (**c**) In contrast, the Apo-A (orange) and Apo-B (yellow-orange) transducer elements superimpose. The Transducer region changes shown here are not as large as those reported for cleft closure as seen in myosin V (Supplementary Fig. 4b) but are following the same trajectory.

OM interactions within the binding cleft include residues of the strands β1 and β2 of the central seven-stranded β-sheet. Comparison of the Apo and OM+ structures reveals changes in the β-sheet that propagates out from the drug-binding cleft and results in a twist of the final three strands (β5, β6 and β7) of the sheet affecting the β-bulge and the HO-β5 loop, key elements of the Transducer region (Fig. 6). The conformations of the two molecules in the OM+ structure (OM-A and OM-B) are not identical to one another in this region (Fig. 6a,b). The OM-B conformation has a greater twist, an extension of the β6 and β7 strands of the sheet into the β-bulge, and displacements of 1–3 Å in the Cα positions for residues of the β-bulge and the HO-β5 loop. There also is subtle shift at the transition of β5-strand into SW-II (arrow in Fig. 6b). The conformations of these elements in both molecules of the Apo structure are essentially identical and differ from both molecules in the OM+ structure (Fig. 6c). The nucleotide-free conformation of the Transducer region of the OM-B molecule is in fact more similar to that in the cardiac myosin MD with AMPPNP bound^14^ than either Apo structure or the OM-A molecule in the same unit cell (Supplementary Fig. 4b).

The crystallographic packing between the two molecules of the Apo and the OM+ structures is essentially identical and does not involve the elements of the Transducer region, suggesting that crystal packing is not the source of this highly localized conformational change. In addition, this region of the structure is well defined in both the Apo and OM+ structures with B-factors that are about half of the average for the protein as a whole. All of these changes are ∼35 Å distant from the OM-binding site and thus are allosteric changes affecting a link in the communication between the nucleotide pocket and the actin-binding interface.

We conclude that the stabilization of the coupling between the SH1 helix, relay helix and converter domain within the drug-binding cleft and the allosteric distortion of the β-sheet in the Transducer region are the principal structural changes associated with drug binding. These changes may be sufficient to account for the shift in the hydrolysis equilibrium in the nucleotide pocket and the accelerated rate of γ-phosphate release contributing to the measured kinetic effects of drug binding on cardiac myosin^6^.

## Discussion

Here we report the structural basis for the allosteric mechanism of the novel cardiac myosin-specific drug, OM. The drug binds to the human β-cardiac myosin motor domain in a narrow cleft between the N-terminal 25-K domain and the lower portion of the 50-K domain. Within the binding cleft the drug is intimately involved with the key elements of the recovery stroke. In addition, OM binding induces allosteric changes distant from the binding cleft that affect the twist of the central seven-stranded β-sheet of the motor domain. These interactions help explain how the drug modulates cardiac myosin motor activity.

In addition to OM, there are several other reversible allosteric effectors of myosin activity that bind to the motor domain including blebbistatin, halogenated pseudilins and EMD 57033. Blebbistatin binds at the apex of the myosin cleft separating the upper and lower 50-K domains blocking the myosin in a product complex with low actin affinity^21^,^22^. The halogenated pseudilins are a class of myosin inhibitors with activity similar to blebbistatin that also bind in the cleft but at a site that is ∼7.5 Å from the blebbistatin site^23^,^24^. EMD 57033 is a thiadiazinone derivative that stabilizes myosin against thermal denaturation and is an allosteric activator of β-cardiac myosin ATPase and myofilament contraction^25^. *In silico* docking studies suggest that it binds in a pocket near the N-terminal SH3 domain. None of these other allosteric effectors bind in or near the OM cleft or are under consideration for therapeutic potential.

**Figure 7:**
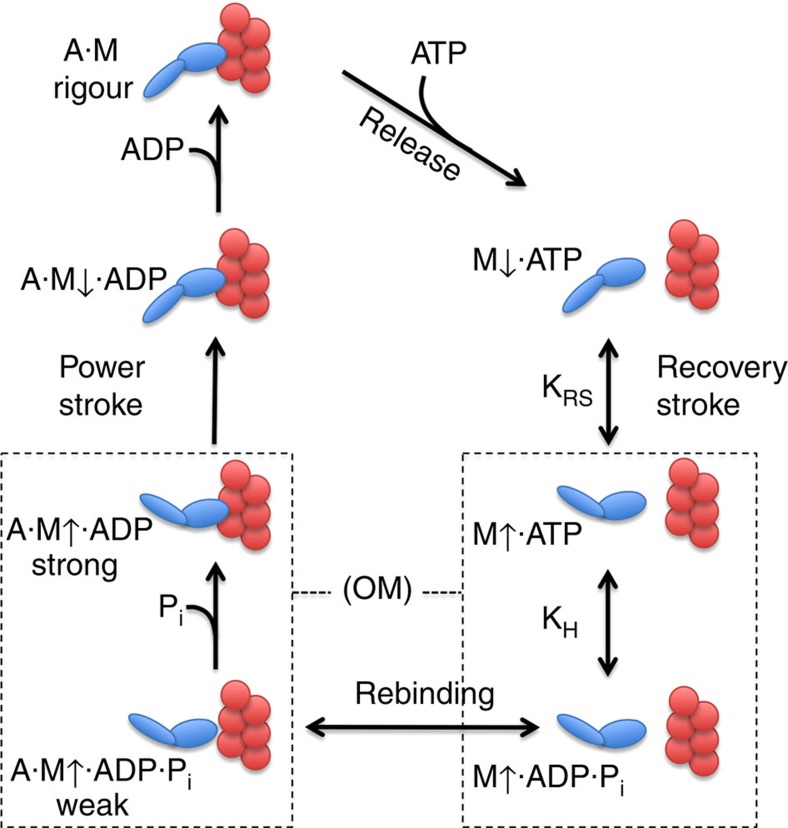
A general kinetic scheme for the actomyosin ATPase cycle. The myosin motor domain (M, blue) is shown at the top left at the end of the cycle, tightly bound to actin (A, red) in the nucleotide-free rigour state (A·M). ATP binds rapidly and releases the MD from actin. The MD then undergoes a conformational equilibrium before ATP hydrolysis between two states designated as M↓ and M↑. These states correspond to the near-rigour (M↓) and pre-power stroke (M↑) conformations. The arrows indicate the down position of the LAH (M↓) and the up position in the pre-power stroke state (M↑) and correlate with intrinsic fluorescent states. The ‘Recovery Stroke' is the rapid equilibrium (*K*_RS_) between these states. The hydrolysis equilibrium (*K*_H_) is coupled to this transition before binding to actin^6^. On rebinding to actin the weak to strong transition is coupled to γ-phosphate (P_i_) release. This results in a conformation change triggering rotation of the LAH producing the ‘Power Stroke' and force production. Rapid ADP dissociation follows, completing the cycle. OM influences the hydrolysis equilibrium (*K*_H_), and it accelerates P_i_ release associated with the weak to strong actin-binding transition^6^. These steps are boxed with dotted lines.

The key to the actomyosin mechanochemical cycle is the ability of the myosin motor domain to couple small changes in the catalytic ATPase site to large conformational changes in both the actin-binding and force-generating domains^26^. This pathway is summarized in the actomyosin kinetic scheme in Fig. 7. The nucleotide bound in the active site (ATP, ADP·P_i_ or ADP) defines the conformation of the MD and its affinity for actin. This requires a communication pathway to amplify changes in the nucleotide pocket and couple them to domain movements. OM acts along this pathway as a traditional allosteric effector binding ∼35 Å from the nucleotide pocket and altering the ATP hydrolysis mechanism and motor activity of cardiac myosin^4^,^6^,^27^. A detailed analysis of the transient kinetics of porcine ventricular HMM reveals that OM does not activate of the actomyosin ATPase activity as was originally suspected^4^ and instead inhibits the *V*_max_ by two- to threefold^6^. This was shown here for the human β-cardiac myosin as well. The principal effect of OM on the myosin kinetic mechanism is a shift in the equilibrium for ATP hydrolysis in the active site towards product formation (M↑̇ADṖP_i_) that is coupled to an increase in the rate of the fast phase of phosphate release. These changes produce an increase in the flux of the intermediates through the actomyosin mechanochemical pathway leading to a faster transition of weak to strong bound actin states. In the kinetic assays, we measured the unloaded rate of ADP dissociation from actomyosin ADP at the end of the contractile cycle and showed that OM does not affect this step. Faster phosphate release without a change in ADP dissociation leads to a higher fraction of myosin bound to actin in a strong ȦM↑̇ADP state^6^.

OM produces a large (15–20-fold) reduction in the unloaded shortening velocity measured in the *in vitro* motility assay (Fig. 2). The reported *K*_i_ reflects the apparent affinity of OM for a composite of steps in the kinetic pathway rather than a binding constant for any single step. Specifically, OM affects both the hydrolysis equilibrium (M↑̇ATP to M↑̇ADṖP_i_) and P_i_ release (AM↑̇ADṖP_i_ to AM↑̇ADP transitions). Thus, the measured *K*_i_ reflects the interaction of OM with an ensemble of states. The effect on the motility assay is likely a result of the recruitment of crossbridges to the actin filament as a consequence of the faster transition from weak to strong bound actin states, resulting in a higher fraction of cMDs tightly bound to actin producing an internal drag on filament movement. Although this slows the velocity of filament movement, it also leads to more force production per filament, a favourable outcome for improving cardiac systolic function. A strain-dependent slowing of the ADP dissociation rate has recently been reported for porcine β-cardiac myosin, and this additional mechanism could contribute to the decrease in filament velocity^28^. A strain-dependent step would extend the duration of systole as is reported in animal models of heart failure treated with OM^5^. An increase in strongly attached crossbridges induced by OM also could facilitate systolic Ca^2+^ activation in the heart by triggering movement of the Tpm-Tn switch out of the inhibitory state. However, the significance of this activation mechanism under physiological conditions in heart muscle has been questioned recently^29^,^30^.

The communication pathway linking the ATPase state to the rotation of the lever arm has been delineated by crystal structures of non-cardiac myosin motor domains with different bound ATP analogues^13^,^18^,^31^,^32^. These structures reveal distinct orientations of the converter domain that lead to an ∼65° rotation of the LAH. On the basis of these known structural states, mutational analyses and molecular dynamics simulations, it has been proposed that with ATP bound the myosin motor domain exists in two alternative conformations, the pre-recovery M↓ state (down state) and post-recovery M↑ state (up state with rotated LAH)^33^,^34^,^35^,^36^,^37^. The transition between these myosin conformations is controlled by the myosin nucleotide interaction and is called the recovery stroke. The pre-recovery state (M↓) has nucleotide bound with SW-II in the open position. In the M↑ state the SW-II loop closes and a glycine in the loop makes a hydrogen bond with the nucleotide γ-phosphate. This small change is transmitted to the coupling region where concerted domain movements result in the rotation of the LAH into the primed, pre-power stroke conformation. In myosin, ATP hydrolysis is a reversible equilibrium in the active site that occurs predominantly in the post-recovery M↑̇ATP state^38^. This prevents unproductive release of hydrolysis products from a pre-recovery state (M↓̇ATP). We have shown that OM specifically shifts the hydrolysis equilibrium towards products (ADṖP_i_). This in turn shifts the coupled equilibria (*K*_RS_ and *K*_H_, Fig. 7) towards the primed LAH conformation (M↑̇ADṖP_i_) for rebinding to actin^6^.

The SW-II loop is contiguous with N terminus of the relay helix providing a link from the nucleotide pocket to the SH1 helix and the converter domain. A defining feature of the M↑ primed conformation is a kink in the relay helix around F489 of the human cMD. On the transition from M↓ to M↑, the bend in the relay helix is strongly coupled to the position of the SH1 helix and the converter domain^13^,^33^,^37^,^39^. The converter domain remains anchored to the relay helix as the converter–LAH undergoes its sweeping rotation. OM binds in the centre of the region that couples the SH1 helix, relay helix and converter domain and involves the key residues linking the SH1 helix to the relay helix (M493-C705 sulfhydryl H-bond) and the relay helix to the converter domain (E497-R712 salt bridge). OM binding shields these interactions from solvent and strengthens the links responsible for the concerted domain movement of the recovery stroke.

An extensive hydrophobic cluster involving Y501, I506, W508, F510, I511, F513, F709 and F764 is also involved in coupling the relay helix and loop with the SH1 helix and the converter domain^33^. The E497-R712 salt bridge lies immediately below this aromatic cluster and is shielded from solvent on three sides by it; thus, OM binding completes the burial of the salt bridge in a hydrophobic pocket. This salt bridge is found in both the near-rigour and pre-power stroke conformation of the scallop myosin II MD structure, suggesting that it is essential to the coupling mechanism^31^. We have shown here that the drug-binding interactions are maintained through a 15° rotation of the converter domain and LAH (∼25% of the recovery stroke), suggesting that the drug may stabilize this coupling region throughout the recovery stroke.

Both E497 and R712 have been identified as sites of cardiomyopathy-associated mutations: an E497D mutation results in a benign hypertrophic cardiomyopathy (HCM) and a R712L mutation is associated with a severe HCM phenotype and sudden cardiac death^40^,^41^. Three of the eight aromatic residues involved in the hydrophobic cluster are also sites associated with HCM (Y501, F513) and DCM (F764) mutations. Five of the sixteen residues that form the drug-binding cleft and interact with OM (M493, E497, V698, P710 and R712) are sites of missense mutations associated with human cardiac pathology. The clustering of cardiomyopathic mutations in this region correlates with the importance of these links to the communication pathway between the nucleotide state and priming of the LAH.

The Transducer is a structural element of the motor domain near the nucleotide-binding site that includes the last three strands of the central seven-stranded β-sheet of the MD^13^. Distortion of the β-sheet is essential for the domain movements needed to accommodate closure of the cleft between the upper and lower portions of the 50-K domain on strong binding to actin^13^,^20^. That in turn reverses the rotation of the LAH leading to the power stroke (Fig. 7). The movements involve a twist of the last three strands (β5, β6 and β7) of the β-sheet, changes in the loops linking β6 to β7 (β-bulge) and the HO helix to β5, and Loop 1 (204–210). It has been suggested that the twist provides the structural basis for communication between the actin interface and the nucleotide pocket and may be involved in the γ-phosphate release^13^,^20^,^39^,^42^. We have found that OM binding alters the conformation of the β-bulge and HO-β5 loop and the twist of the β5, β6 and β7 strands when compared with the Apo structure and between the two cMD conformations within the OM+ structure. These structural changes are consistent with a mechanism, whereby OM accelerates the rate of P_i_ release and the weak to strong actin-binding transition by facilitating β-sheet distortion.

We conclude that OM binding acts to stabilize the interactions within the coupling region. This region is central to the recovery stroke and the priming of the cMD for a productive contractile cycle. In addition, drug binding shifts the conformational equilibrium of the Transducer region to favour phosphate release. Together, these subtle changes alter the mechanical tuning of β-cardiac myosin producing changes in activity that in cardiac muscle will increase force production at the expense of speed of contraction. The candidate drugs from which OM was developed were originally identified by a high-throughput screen of a biochemically reconstituted contractile system^3^. That screen and subsequent selection of OM has identified a fundamentally important site for fine-tuning the motor activity of myosin.

## Methods

### Construction of the β-cardiac myosin MD expression vector

A cDNA encoding the human β-cardiac muscle myosin HMM was kindly provided by James Sellers (National Heart, Lung, and Blood Institute (NHLBI)) from a clone isolated by Giovanni Cuda and Neil Epstein (NHLBI). Construction of the β-cardiac MD::GFP chimera is based on the skeletal muscle MD::GFP chimera (S1_795_GFP) described in detail elsewhere^9^. A cDNA encoding residues 1–787 of the cardiac myosin motor domain was amplified with PCR using primers to insert a unique NotI site at the 5′ end of the open reading frame and a unique MluI site at the 3′ end. The cardiac MD sequence then replaced the skeletal muscle myosin MD in the shuttle vector pSH-S1_795_GFP (ref. 9). A four-residue linker (788–791) forms the fusion site to the GFP-coding sequence that comprise residues 5–238 of *A. victoria* GFP. The complete coding region was confirmed by sequencing. A FLAG-tagged variant of chimeric protein was prepared by mutating the C-terminal-coding sequence of the GFP domain from DELYK to DYKDHD. This minimal FLAG tag sequence added a single additional residue to the protein, with no effect on protein activity or crystallization conditions^43^.

### Adenovirus manipulation

Recombinant adenovirus DNA was prepared by homologous recombination of pSH-CaMD::GFP with the pAdEasy1 vector in *E. coli* strain BJ5183 (refs 9, 12, 44). Colonies were selected for kanamycin resistance and plasmid DNA was characterized by restriction digestion. The recombinant virus DNA was transformed into *E. coli* DH10B cells, and the purified pAdCaMD DNA was linearized by digestion with PacI and transfected into human 293 cells (CRL 1573; American Type Culture Collection, Rockville, MD) for adenovirus packaging and amplification^44^. The human 293 cells are maintained in growth medium containing DMEM with 10% fetal bovine serum (FBS), 1 mM sodium pyruvate and 0.5% gentamycin at 37 °C and 5% CO_2_. Cells were monitored using fluorescence microscopy for expression of the cMD::GFP fusion protein. The original human β-cardiac HMM clone encodes residues 1–1,138 of the *MYH7* gene with a FLAG tag added on the C terminus (1,139–1,146) of the S2 domain. The cHMM cDNA was cloned into the pShuttle-IRES-hrGFP-1 vector (Stratagene, LaJolla CA), and an AdcHMM-Flag virus was prepared and amplified for expression and purification of the cHMM protein in C2C12 cells. The virus was expanded by infection of a large number of plates of confluent 293 cells at an multiplicity of infection of 3–5. The virus was harvested from the cells by cycles of freeze-thaw, and then it was purified by CsCl density sedimentation yielding final virus titres of 10^10^–10^11^ plaque-forming units per ml (p.f.u. ml^−1^).

### Muscle cell expression and purification of β-cardiac MD::GFP

Myoblasts of the mouse C2C12 cell line (CRL 1772; American Type Culture Collection) are maintained by seeding the cells at an initial density of 7.5 × 10^4^ cells cm^−2^ in 90% DMEM, 10% FBS and passaging the cells at less than 60% confluence^9^,^12^,^45^. Confluent C2C12 myoblasts are infected with replication-defective recombinant adenovirus (Ad-CaMD::GFP) at 5 × 10^8 ^p.f.u. ml^−1^ in fusion medium (89% DMEM, 10% horse serum, 1% FBS) to induce differentiation. Expression of recombinant cMD::GFP is monitored by accumulation of GFP fluorescence in infected cells. Myocyte differentiation and fluorescence accumulation are monitored for the next 120–144 h when the cells are harvested. Cells are chilled, media is removed and the cell layer is rinsed with cold PBS. The cells are scraped into 0.5 ml per dish of Triton extraction buffer: 100 mM NaCl, 0.5% Triton X-100, 10 mM imidazole pH 7.0, 1 mM dithiothreitol (DTT), 5 mM MgATP and Protease Inhibitor cocktail (Sigma, St Louis). The cell suspension is collected in an ice-cold Dounce homogenizer and lysed with 15 strokes of the A pestle. The cell debris in the whole-cell lysate is pelleted by centrifugation at 17,000*g* for 15 min at 4 °C. The Triton-soluble extract is fractionated by ammonium sulfate precipitation using two sequential steps of 0–30% saturation and 30–60% saturation. The CaMD::GFP precipitates between 30 and 60% saturation of ammonium sulfate.

The recovered pellet is dissolved in 1/10 the original volume of buffer and dialysed against 25 mM imidazole, pH 7.0, 1 mM DTT for anion exchange chromatography of the cMD lacking the FLAG tag sequence, or into the same buffer without DTT for affinity purification of the FLAG-tagged cMD on M2 monoclonal antibody-Sepharose beads (Sigma). Anion exchange chromatography on a HR 5/5 Mono-Q column at 23 °C (Pharmacia Biotech, Piscataway, NJ) was carried out as previously described^9^. The FLAG-tagged cMD was bound to an M2-Sepharose column, washed and eluted with 0.1 mg ml^−1^ FLAG peptide (Sigma). The FLAG-tagged human cHMM was purified in the same manner as the tagged cMD. Protein was concentrated and buffer-exchanged on Amicon Ultracel-10 K centrifugal filters (Millipore; Darmstadt, Germany), checked by SDS–PAGE (Supplementary Fig. 1), aliquoted and stored at −80 °C.

### Motility and ATPase assays

An anti-GFP monoclonal antibody (3E6; Molecular Probes; Grand Island, NY) was bound to freshly prepared nitrocellulose-coated glass coverslips^9^. The cMD at 25 μg ml^−1^ was incubated with the surface and bound via the GFP domain as previously described. Skeletal muscle myosin was bound to the surfaces via an anti-S2 monoclonal antibody^46^,^47^. Recombinant human β-cardiac HMM at 40 μg ml^−1^ was bound directly to fresh nitrocellulose surfaces as described^6^,^46^. The surfaces were blocked with 1% bovine serum albumin in PBS for 5 min. Motility is measured in a 12-μl assay chamber in motility buffer (25 mM Imidazole, 25 mM KCl, 4 mM MgCl_2_, 7.5 mM Mg^2+^ATP, 0.5% methyl cellulose, 0.1 mg ml^−1^ glucose oxidase, 0.018 mg ml^−1^ catalase, 2.3 mg ml^−1^ glucose and 5 mM 2-mercaptoethanol, pH 7.6) containing 1 nM phalloidin–rhodamine-labelled actin. OM (CK-1827452) was purchased from Selleckchem.com (Boston, MA). To titrate the effect of the drug on motility, a 2.5-mM stock of OM in dimethylsulphoxide (DMSO) was serially diluted with DMSO before a final 1/200 dilution into motility buffer with the rhodamine-labelled actin. The final 0.5% DMSO in the assay buffer had no effect on motility in the absence of drug. The chamber is observed with a temperature-controlled stage and objective set at 32 °C on an upright microscope with an image-intensified charge-coupled device camera capturing data to an acquisition computer at 5–30 f.p.s. dependent on assay parameters. Movement of actin filaments from 500 to 1,000 frames of continuous imaging is analysed with semiautomated filament tracking programmes^12^,^46^,^48^. The trajectory of every filament with a lifetime of at least 10 frames is determined; the instantaneous velocity of the filament moving along the trajectory, the filament length, the distance of continuous motion and the duration of pauses are tabulated. A weighted probability of the actin filament velocity for hundreds of events is fit to a Gaussian distribution and reported as a mean velocity and s.d. for each experimental condition.

Steady-state ATPase activity was measured as described previously^49^. Experiments were carried out in a buffer containing 4 mM MOPS, 2 mM MgCl_2_ with or without 100 μM OM (0.5% DMSO), pH 7.2. The cMD at 0.06 μM was used to measure the ATPase activity over a range of actin (0–100 μM). The ATPase activity of the actin filaments alone was subtracted from the actomyosin data.

### Crystallization and structure determination

Human β-cardiac MD::GFP protein (1.5–2.0 mg ml^−1^) in 25 mM HEPES pH 7.0, 5 mM TCEP (Sigma) was screened against four Hampton Research (Aliso Viejo, CA) high-throughput crystallization screens using 0.2 μl protein and 0.2 μl mother liquor (ML) per drop equilibrated against 60 μl of ML at room temperature. Five drops containing brightly fluorescent birefringent crystals were found. These initial conditions were refined and single crystals without drug (Apo) were grown from 10% Tacsimate, pH 6.0, 14–15% PEG 3350, 10% glycerol, 0.2 mM MgCl_2_ and 5 mM TCEP. Crystals with OM (OM+) were grown in essentially the same condition with 125 μM OM added to the protein drop (0.5% DMSO). The crystallization conditions proved to be a suitable cryoprotectant; therefore, crystals taken directly from the ML were fast-cooled in liquid nitrogen for X-ray analysis.

Single-crystal diffraction data for the Apo structure were collected on a Rigaku rotating anode X-ray generator (*λ*=1.542 Å) with a Raxis IV++ detector at 100 °K. The OM+ crystal diffraction data were collected at Brookhaven National Laboratory NSLS beamline X29A (*λ*=1.075 Å) with an ADSC QUANTUM 315 detector at 100 °K. Reflections were indexed, integrated and scaled with iMosflm and Aimless from the ccp4 package^50^,^51^. The Apo structure was determined first by molecular replacement with Phaser^52^ using residues 4–780 of chicken skeletal muscle myosin subfragment 1 structure, PDB ID: 2MYS^15^, together with the structure of GFP, PDB ID: 2QLE^53^, as the search ensemble. The molecular replacement map clearly identified two motor domains associated with one GFP per domain. The helix linking the MD to the GFP was not included in the search models; however, new density corresponding to the helix was apparent in the initial maps. After building the linking helix into the density and joining the MD and GFP domains, the cMD and GFP sequence were built into the model with PHENIX Autobuild^54^. The structure was refined with PHENIX refine^55^ and iterative model building in Coot^56^. Only a very limited number of well-defined water molecules and two sulfate anions in the cMD β-phosphate-binding loop were included in the structure at this resolution. The statistics for favoured, allowed and outlier Ramachandran angles are 93.4%, 6.1% and 0.5%, respectively. The Molprobity score for the refined structure is 2.16 and the coordinates are deposited as PDB ID: 4P7H^57^,^58^.

The OM+ structure was solved by molecular replacement using the motor domain (2–708) of the Apo structure A chain and the helix–GFP domain as separate models in the search ensemble followed by PHENIX Autobuild. After limited initial refinement, unassigned density likely coming from the ligand was apparent. PHENIX LigandFit was used to search and dock OM into the map using Resolve^59^. Ligand restraints for refinement were generated with eLBoW^60^. The structure was refined as with the Apo structure. The statistics for favoured, allowed and outlier Ramachandran angles are 95.3%, 4.2% and 0.5%, respectively. The Molprobity score for the refined structure is 1.87.

The Karplus and Diederichs CC* data and model quality-assessment tool were applied to both models and data sets to assess the high-resolution cutoff^61^. The Apo data set was truncated at 3.2 Å, with mean (*I*/*σ*(*I*))=2 and a CC*>0.88 for the highest-resolution shell (3.3–3.2 Å). The CC* statistic for the OM+ data is >0.73 for the highest-resolution shell (2.33–2.25 Å), supporting the inclusion of the full data range in determination of the model^62^,^63^. Alternatively, applying traditional cutoff criteria of the mean (*I*/*σ*(*I*))≥2 to the OM+ data would make this a nominal 2.40-Å resolution structure. The coordinates for the OM+ structure are deposited as PDB entry 4PA0 (ref. 57). Data collection and refinement statistics are summarized in Table 1.

## Additional information

**Accession codes:** Coordinates and structure factors for Apo cMD and OM + cMD have been deposited in the Protein Data Bank under accession codes 4P7H and 4PA0 respectively.

**How to cite this article:** Winkelmann, D. A. *et al*. Structural basis for drug-induced allosteric changes to human β-cardiac myosin motor activity. *Nat. Commun.* 6:7974 doi: 10.1038/ncomms8974 (2015).

## Supplementary Material

Supplementary Information Supplementary Figures 1-5, Supplementary Tables 1-2 and Supplementary References

## Figures and Tables

**Table 1:** Data collection and refinement statistics.

	Apo cMD (4P7H)*	**OM+ cMD (4PA0)**†
*Data collection*		
Space group	P1	P2_1_
Cell dimensions		
*a*, *b*, *c* (Å)	59.6, 97.7, 118.1	100.2, 88.9, 137.8
α, β, γ (°)	71.0, 82.4, 75.0	90.0, 93.9, 90.0
Resolution (Å)	25.0–3.2 (3.3–3.2)‡	46.0–2.25 (2.33–2.25)
*R*_merge_	0.352 (1.299)	0.258 (1.367)
*I*/*σ*(*I*)	9.0 (2.0)§	9.0 (1.1)§
Completeness (%)	99.5 (98.0)	93.3 (84.1)
Redundancy	11.3 (10.2)	7.4 (3.3)
		
*Refinement*		
Resolution (Å)	3.2	2.25
No. of reflections	39,840 (3,966)	107,033 (9,608)
*R*_work_/*R*_free_	0.191/0.265	0.201/0.246
No. of atoms		
Protein	14,882	14,533
Ligand/ion	54	118
Water	5	306
*B*-factors		
Protein	49.0	46.0
Ligand/ion	59.8	62.3
Water	24.2	33.8
R.m.s.d.		
Bond lengths (Å)	0.007	0.011
Bond angles (°)	1.08	1.35

cMD, β-cardiac motor domain; OM, Omecamtiv Mecarbil; r.m.s.d., root-mean-square deviation.

^*^Data collected from three crystals.

^†^Data collected from a single crystal.

^‡^Values in parentheses are for the highest-resolution shell.

^§^The Apo data set was truncated at 3.2 Å with the mean (*I*/*σ*(*I*))=2 and a CC*>0.88 for the highest-resolution shell^61^. The CC* statistic for the OM+ 2.33–2.25 Å resolution shell is >0.73, supporting the inclusion of the full data range in determination of the model^62^,^63^. Applying traditional cutoff criteria of the mean (*I*/*σ*(*I*))≥2 would make this a nominal 2.40-Å-resolution structure.
