# Supplementary material for: Structural basis for drug-induced allosteric changes to human β-cardiac myosin motor activity
Source: Nat Commun. 2015 Aug 6;6:7974. doi: 10.1038/ncomms8974 (PMC4918383; doi:10.1038/ncomms8974)
Supplement: Supplementary Information — Supplementary Figures 1-5, Supplementary Tables 1-2 and Supplementary References [file ncomms8974-s1.pdf]

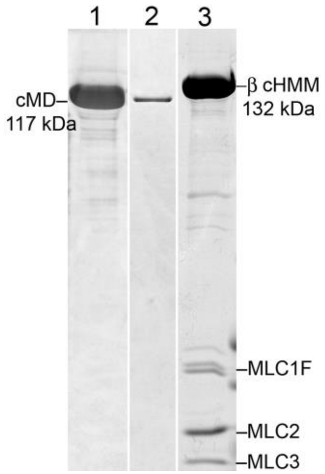

MS/MS Mass Spectrometry analysis of crystallized protein

log(e)[1]	log(I)[2]	%/%[3]	#[4]	Total[5]	Mr[6]	Identified Match
-2290.6	7.97	62/100+	172	709	116.3	cMD::GFP Chimeric Protein
-1236.7	7.83	22/34	104	506	222.7	Human *MYH7* β-cardiac myosin
-258.2	6.89	37/58	23	71	26.9	*A. victoria* GFP

[1]Probability (expressed as $\log_{10}$) that this expectation is stochastic
[2]Sum of the intensities of the fragment ion spectra expressed as $\log_{10}$
[3]Protein Coverage: % of protein residues/% corrected for unlikely peptides
[4]Number of unique peptides found
[5]Total number of peptides found from this sequence
[6]Protein molecular mass in kDa

Supplementary Figure 1. Biochemical analysis of purified cMD::GFP chimeric protein and human β-cHMM. Purified proteins used in biochemical assays and for structure determination were analyzed by SDS PAGE: Lane 1, purified cMD::GFP; Lane 2, four isolated single cMD crystals (+OM), washed and re-dissolved; Lane 3, human β-cHMM used in motility assay. The single 117 kDa cMD band from Lane 2 (crystals) was extracted, trypsin digested, and analyzed by MS/MS spectroscopy. The MS/MS analysis confirms sequences are from the human *MYH7* and *A. victoria* GFP genes. The coverage of peptides is essentially complete when compared to the sequence of the chimeric gene in the expression vector.

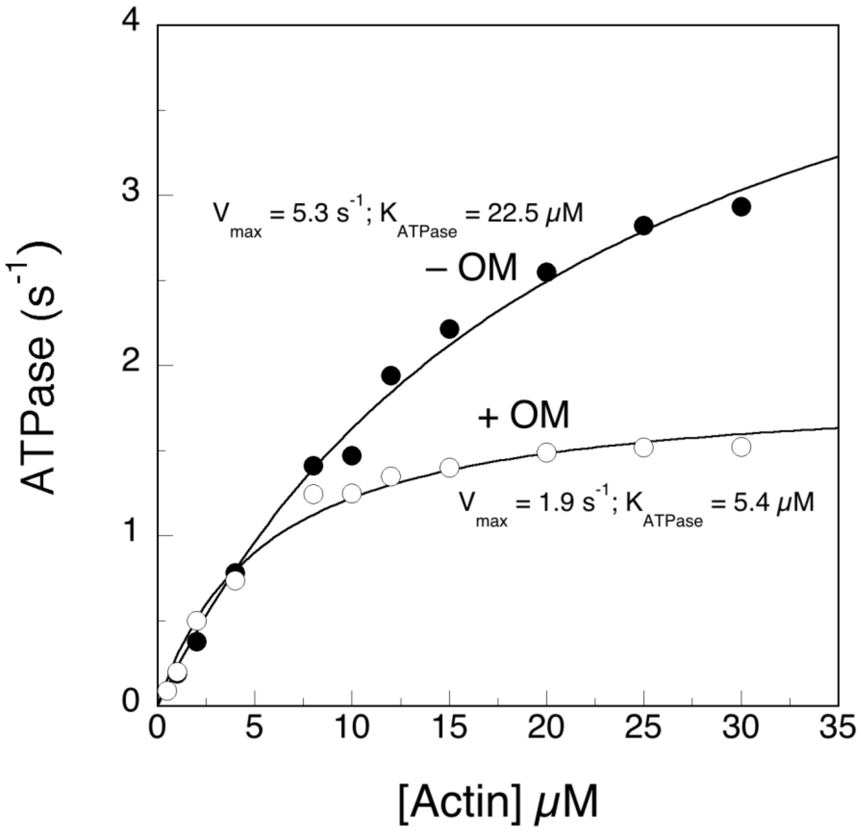

Supplementary Figure 2. OM inhibits the V_{max} of the actin-activated ATPase assay of human β-cardiac MD and it reduces the K_{ATPase} for actin 4 fold. The steady-state ATPase rates were measured with an NADH-coupled assay at 25°C. The ATPase rate of cMD (0.06 µM) was assayed over a range of actin filament concentrations (0-30 µM) with or without 100 µM OM. The rates were corrected for the basal ATPase activity of actin alone and expressed as turnover numbers (s^{-1}). For comparison, the V_{max} for porcine ventricular HMM decreased from 2.5 s^{-1} to 1.3 s^{-1} with OM and the K_{ATPase} decreased from 6 µM to 3.1 µM.

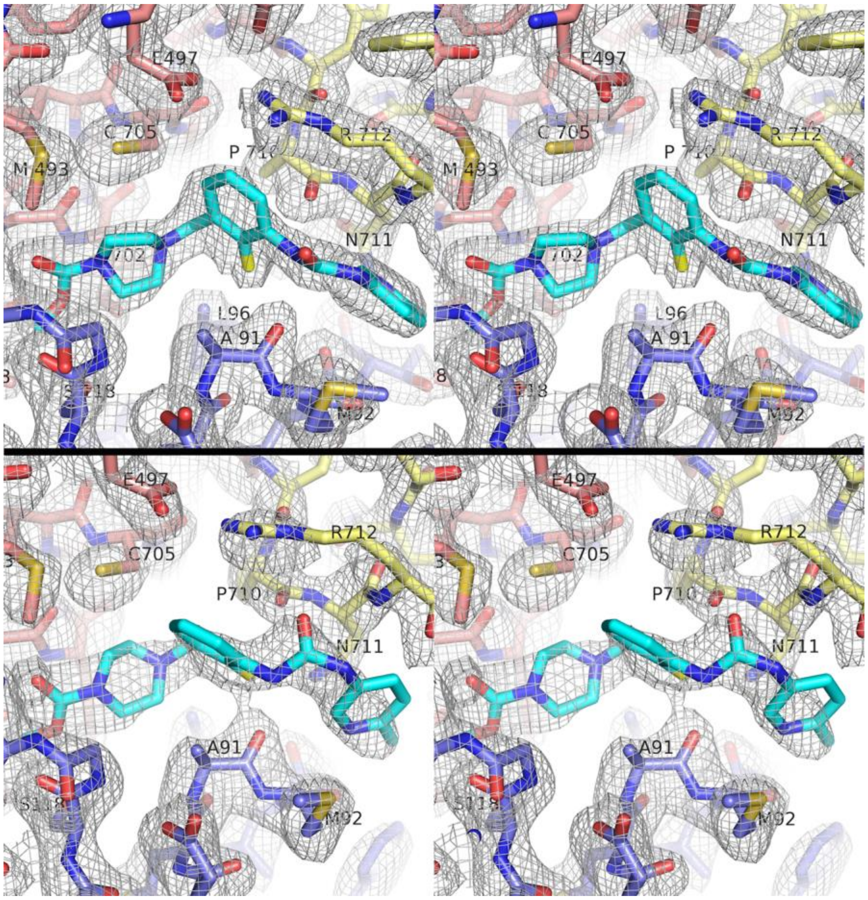

Supplementary Figure 3. Portions of the electron density map of the OM+ structure. The electron density around the OM binding cleft of the A-chain is shown in stereo superimposed on the model in the **upper panel** and the OM binding cleft of the B-chain in the **lower panel**. Both views are centered on the fluoro-benzine ring of OM with the fluorine atom colored yellow. The 2Fo-Fc map is displayed contoured at 1σ threshold. Residues interacting with the drug are labeled. The view in the **upper panel** is similar to the A-chain orientation in Figure 3a, and the corresponding region of the B-chain is shown in the **lower panel**.

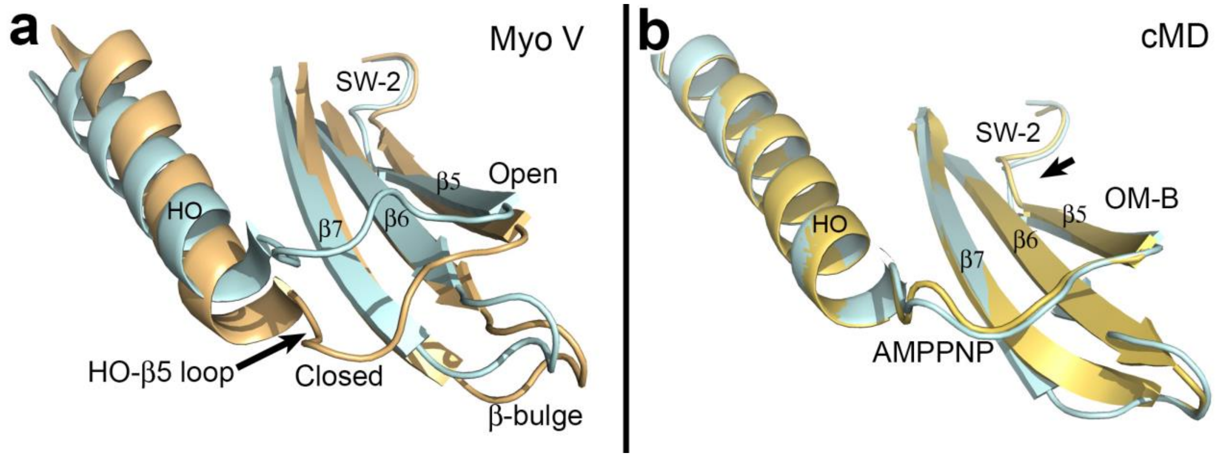

Supplementary Figure 4. Illustration of the twist in the Transducer elements between molecules in the myosin V and human β-cardiac myosin structures. **a.** The Transducer elements were first described in myosin V (Myo V) by comparison of two nucleotide free structures, one with an Open cleft (1W7J; cyan) and a second with a Closed cleft (1OE9; orange) [5]. Distortion of strand β5-β7 and the associated loops and linkers are coupled to rotation of the upper 50K domain around the HO helix. The Myo V changes involve full cleft closure and are more dramatic than those seen for OM binding where the cleft between the upper and lower 50K domain remains open. The pseudo-atomic model derived from the 8 Å EM structure of actin-tropomyosin-myosin I complex has confirmed the conformational changes described in Myo V as the rigor state with cleft closure and twisting of the central β-sheet [6]. **b.** The structure of the β-cardiac myosin has been reported with AMPPNP bound in the nucleotide pocket[7]. The nucleotide-free conformation of the Transducer region of the OM-B molecule (yellow-orange) is more similar to that in the cardiac myosin MD with AMPPNP bound (cyan) than either the Apo structure or the OM-A molecule in the same unit cell. The conformation of the nucleotide pocket of OM-B is essential identical to that of the AMPPNP structure as well.

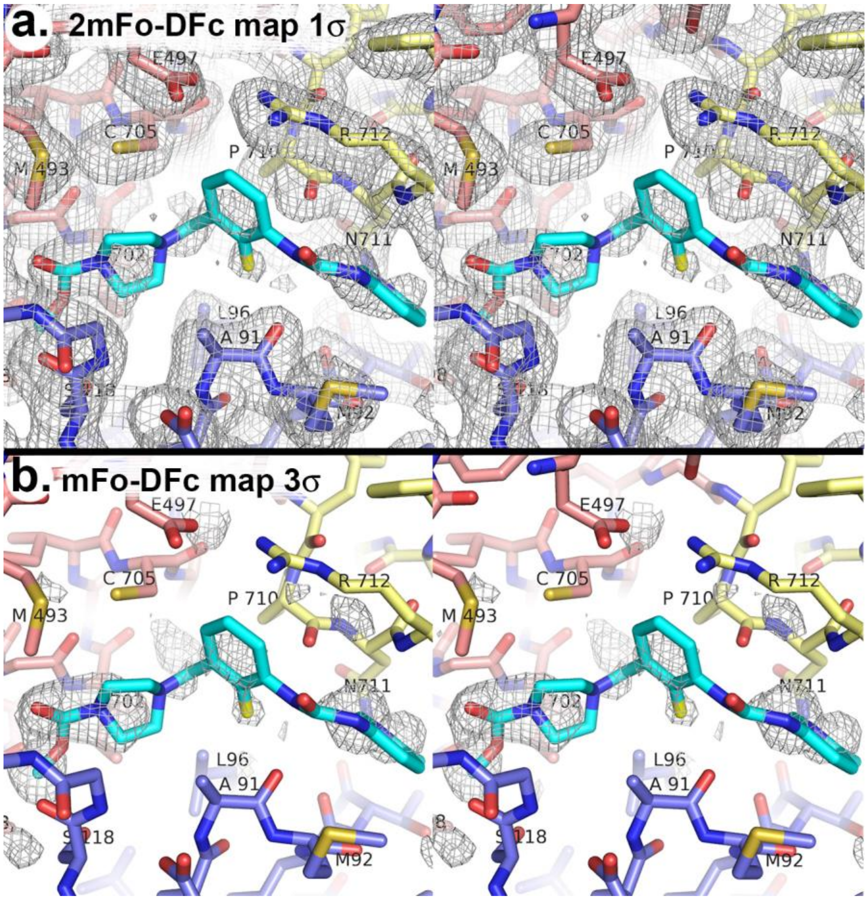

Supplementary Figure 5. Stereo omit maps derived by refinement of the data against a model with both ligands removed and using phases calculated from the OM omit model. Portions of the electron density maps are shown centered on the drug-binding site of the A-chain. Both maps are superimposed on the complete model to show the OM position **a.** The 2mFo-DFc omit map is contoured at 1σ threshold. **b.** The mFo-DFc omit map is contoured at 3σ**.**

Supplementary Table 1: Ligand Binding Interactions

Omecamtiv Mercarbil: methyl 4-(2-fluoro-3-{[6-methylpyridin-3-yl) carbamoyl]amino}benzyl)piperazine-1-carboxylate

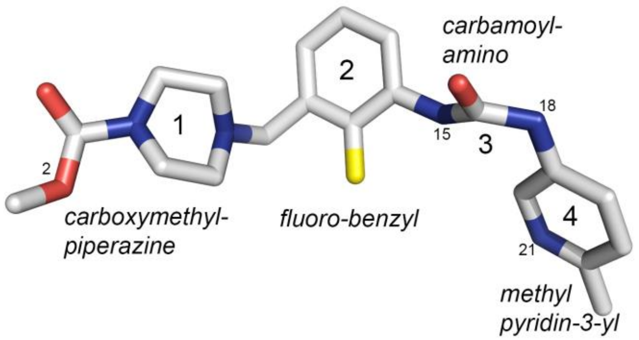

Residue	MD Feature	OM Feature	Interaction Type
	N-Terminal 25 K Domain		
A91	Turn	3	H-bond A91 C=O···H-N_{15} OM (3.1 Å)
M92	Turn	3	H-bond M92* Sδ···H-N_{18} OM (4.2 Å)
L96	Loop	1	Packing
S118	β1–β2 loop	1	H-bond, S118 Oγ-H···O_2 OM (3.4 Å)
G119	β1–β2 loop	1	Packing
F121	β2 strand	1	Packing
	Lower 50 K Domain		
M493	Relay Helix	1	Packing, M493* Sδ···H-Sγ C705 H-bond (4.1 Å)
E497	Relay Helix	1, 2	Packing, Salt bridge with R712
V698	SH1 Helix	1	Packing
G701	SH1 Helix	1	Packing
I702	SH1 Helix	1, 2	Packing
C705	SH1 Helix (SH1)	2	Packing, C705* Sγ-H···Sδ M493 H-bond (4.1 Å)
	Converter Domain		
P710	Turn	3	Packing
N711	β1' strand	4	H-bond N711 Nγ-H···N_{21} OM (3.0 Å)
R712	β1' strand	3, 4	Packing, Salt bridge with E497
L770	β1' strand	4	Packing

The structure and systematic name of the ligand Omecamtiv Mecarbil (OM) is shown. The molecule is divided into 4 sub-regions to facilitate mapping the interactions with the protein side chains, and selected OM atoms are numbered (N_{15}, N_{18}, N_{21} & O_2). Packing interactions on OM binding results in a 40-80% increase in the buried surface area for 12 of 16 residues interacting with the drug [1]. Hydrogen bonds between protein residues and OM are delineated and correspond to the H-bonds illustrated in Figure 3 and Figure 5. *Non-canonical sulfur-containing hydrogen bonds contribute to the structure of the OM binding cleft and interactions [2].

Supplementary Table 2. Myosin sequence conservation in the OM binding cleft

Myosin	25K Domain	β1-β2 Loop	Relay Helix	SH1 Helix	Hinge	CD β1′
Residue #	91 96	118 121	493 497	698 707	708-710	711-713
Hum *MYH7*	AMLTFL	SGLF	MFVLE	VLEGIRICRK	GFP	NRI
Por *MYH7*	------	----	-----	----------	---	---
Bov *MYH7*	------	----	-----	----------	---	---
Rat *MYH7*	------	----	-----	----------	---	---
Rat *MYH6*	------	----	-----	----------	---	---
Chk *N116*	--M---	----	-----	----------	---	S-V

The human β-cardiac myosin sequence (Hum *MYH7*) is compared to the porcine (Por), bovine (Bov), rat (Rat), and the chicken pectoralis muscle myosin (Chk *N116*) sequences. The effects of Omecamtiv Mecarbil on the kinetic and motile activity of human β-cardiac myosin and chicken skeletal muscle myosin are reported here (Fig. 3 and Supplementary Fig. 2). OM has been shown to modulate the activity of the porcine, bovine, and rat cardiac myosin isoforms [3,4]. The sequence of the residues involved in the binding interface (highlighted in yellow) for these cardiac myosin isoforms is identical (‘-‘). The chicken skeletal myosin motor activity is unaffected by OM, and it has three sequence changes among these interface residues. One of these changes, N711 is a serine that potentially weakens a key hydrogen bond between the first β-strand of the converter domain and the nitrogen in the methyl-pyridinyl ring of OM.

Supplementary References

1. Krissinel, E. & Henrick, K. Inference of macromolecular assemblies from crystalline state. *J Mol Biol* **372**, 774-97 (2007).
2. Zhou, P., Tian, F., Lv, F. & Shang, Z. Geometric characteristics of hydrogen bonds involving sulfur atoms in proteins. *Proteins* **76**, 151-63 (2009).
3. Malik, F.I. et al. Cardiac myosin activation: a potential therapeutic approach for systolic heart failure. *Science* **331**, 1439-43 (2011).
4. Liu, Y., White, H.D., Belknap, B., Winkelmann, D.A. & Forgacs, E. Omecamtiv Mecarbil Modulates the Kinetic and Motile Properties of Porcine beta-Cardiac Myosin. *Biochemistry* **54**, 1963-75 (2015).
5. Coureux, P.D., Sweeney, H.L. & Houdusse, A. Three myosin V structures delineate essential features of chemo-mechanical transduction. *EMBO J* **23**, 4527-37 (2004).
6. Behrmann, E. et al. Structure of the rigor actin-tropomyosin-myosin complex. *Cell* **150**, 327-38 (2012).
7. Klenchin, V., Deacon, J., Combs, A., Leinwand, L. & Rayment, I. Cardiac human myosin S1dc, beta isofrom complexed with Mn-AMPPNP. *PDB ID: 4DB1* (2012).
